# The Role of Flavonoids in the Osteogenic Differentiation of Mesenchymal Stem Cells

**DOI:** 10.3389/fphar.2022.849513

**Published:** 2022-04-06

**Authors:** Jinli Zhang, Zhihe Liu, Yang Luo, Xiaojian Li, Guowei Huang, Huan Chen, Aiguo Li, Shengnan Qin

**Affiliations:** ^1^ Guangzhou Institute of Traumatic Surgery, Department of Orthopedics, Guangzhou Red Cross Hospital, Medical College, Jinan University, Guangzhou, China; ^2^ School of Physical Education, Southwest University, Guangzhou, China; ^3^ Department of Burn and Plastic Surgery, Guangzhou Red Cross Hospital, Medical College, Jinan University, Guangzhou, China

**Keywords:** tissue engineering, osteogenic differentiation, mesenchymal stem cells, flavonoids, osteoporosis

## Abstract

Mesenchymal stem cells (MSCs) play an important role in developing bone tissue engineered constructs due to their osteogenic and chondrogenic differentiation potential. MSC-based tissue engineered constructs are generally considered a safe procedure, however, the long-term results obtained up to now are far from satisfactory. The main causes of these therapeutic limitations are inefficient homing, engraftment, and directional differentiation. Flavonoids are a secondary metabolite, widely existed in nature and have many biological activities. For a long time, researchers have confirmed the anti-osteoporosis effect of flavonoids through *in vitro* cell experiments, animal studies. In recent years the regulatory effects of flavonoids on mesenchymal stem cells (MSCs) differentiation have been received increasingly attention. Recent studies revealed flavonoids possess the ability to modulate self-renewal and differentiation potential of MSCs. In order to facilitate further research on MSCs osteogenic differentiation of flavonoids, we surveyed the literature published on the use of flavonoids in osteogenic differentiation of MSCs, and summarized their pharmacological activities as well as the underlying mechanisms, aimed to explore their promising therapeutic application in bone disorders and bone tissue engineered constructs.

## Introduction

People are living longer than ever before because of medical, social and economic advances in the whole world. However, increasing life expectancy also brings tremendous challenges to the society, like chronic non-communicable diseases including osteogenesis are becoming the leading cause of death and disability (Williams et al., 2018; Garmany et al., 2021). Osteoporosis is a condition in that bones become weaker and more fragile owing to bone mass loss with ageing, diseases and drugs, so the chances are higher they’ll crack or break. It is predicted that osteogenesis fractures will account for over 50% of the total fractures, and unlike bone fractures in young people, osteogenesis fractures induce a large proportion of disability and mortality in elderly people (Patel et al., 2021). Additionally, poor fracture healing can cause critical-sized bone defects (Miller, 2016; Nauth et al., 2018). Mesenchymal stem cells (MSCs)are a kind of adult stem cells with multiple differentiation potentials (Friedenstein, 1976) and exist in a variety of tissues including bone marrow, adipose tissue, umbilical cord, etc., (Baksh et al., 2004). Stand as promising candidates in the treatment of bone defects and other degenerative bone diseases, MSCs have great potential use in the bone repair and regeneration owing to their osteogenic differentiation potential and extensive sources. However, the ability of MSC to differentiate into osteoblasts may become impaired under certain pathophysiological conditions including oxidative stress and inflammation (García-Sánchez et al., 2019). Hence, the strategies aimed to increase cell survival and osteogenic capacity are important for the MSCs-based bone regeneration therapies. Strategies, including promoting MSCs osteogenic differentiation through genetic modification (Armstrong and Stevens, 2019), or providing the appropriate extracellular environmental cues like scaffolds, growth factors or other bioactive molecules, are commonly used (Velasco et al., 2015; Yang et al., 2017). For example, combining β-tricalcium phosphate and BMP-2 has been proven to be effective to enhance the osteogenesis of MSCs (Dimitriou et al., 2011).

Some botanical drugs have been effective and safe in the treatment of fracture healing in China for a long time, and more and more evidences show many ingredients of them are beneficial to bone health. Flavonoids are commonly present in botanicals, they are synthesized in plants as secondary metabolites, and characterized with diverse pharmacological properties (Martens and Mithöfer, 2005). Natural flavonoids and their glycosides have been identified and explored for their therapeutic potentials in different fields including osteoporosis-related complication and disorders. Many flavonoids exerted promoting bone formation and anti-osteoporosis effects through stimulating osteogenic differentiation of MSCs (Huang et al., 2018; Wang et al., 2018; Casado-Díaz and Rodríguez-Ramos, 2021). Also, European nutritional studies demonstrated that daily intake of flavonoids contributed to good bone health (Zamora-Ros et al., 2016), Therefore, combining flavonoids and MSCs would be an efficient strategy to enhance bone formation and increase cell survival in the field of bone tissue engineering.

## Basic Structure and Classification of Flavonoids

Flavonoids are a kind of polyphenolic compounds widely present in nature and have spectral biological activities. In terms of chemical structure, flavonoids generally refer to a series of hydroxylated phenolic molecules consisting with a C6-C3-C6 units, in which two benzene rings (A and B rings) are linked to each other through three central carbon atoms ring (ring C). These compounds can be divided into many different classes according to the oxidation degree of the central three carbons, whether the three carbons constitute a ring and the connection site of B ring, and so on. Generally, flavonoids are mainly classified into the following subclasses: flavanones, flavonols, flavonones, and isoflavones, anthocyanins, flavanols, (Amarowicz et al., 2009; Kumar and Pandey, 2013), and their basic chemical structure and representative compounds are shown in Table 1.

**TABLE 1:** Structure of flavonoids subclasses.

Subclass name	Core chemical structure	Typical compounds
Flavanones	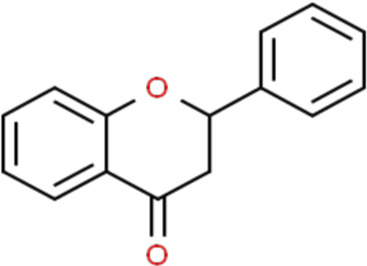	Naringin, Hesperetin
Flavonols	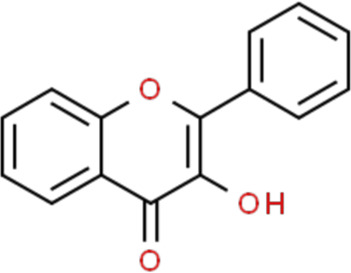	Quercetin, Kaempferol, Rutin
Flavones	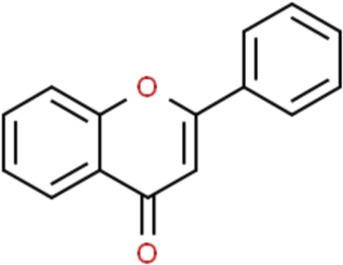	Luteolin, Apigenin
Isoflavones	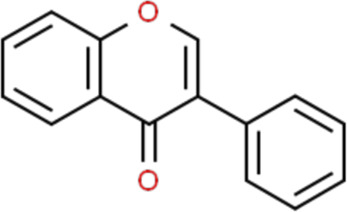	Genistein, Daidzein
Anthocyanins	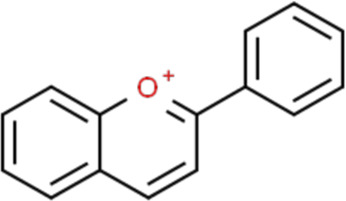	Delphinidin, Cyanidin
Chalcones	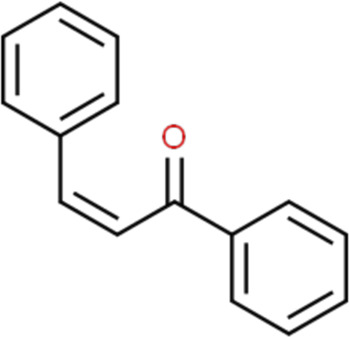	Xanthohumol

## Biological Activity of Flavonoids

**FIGURE 1:**
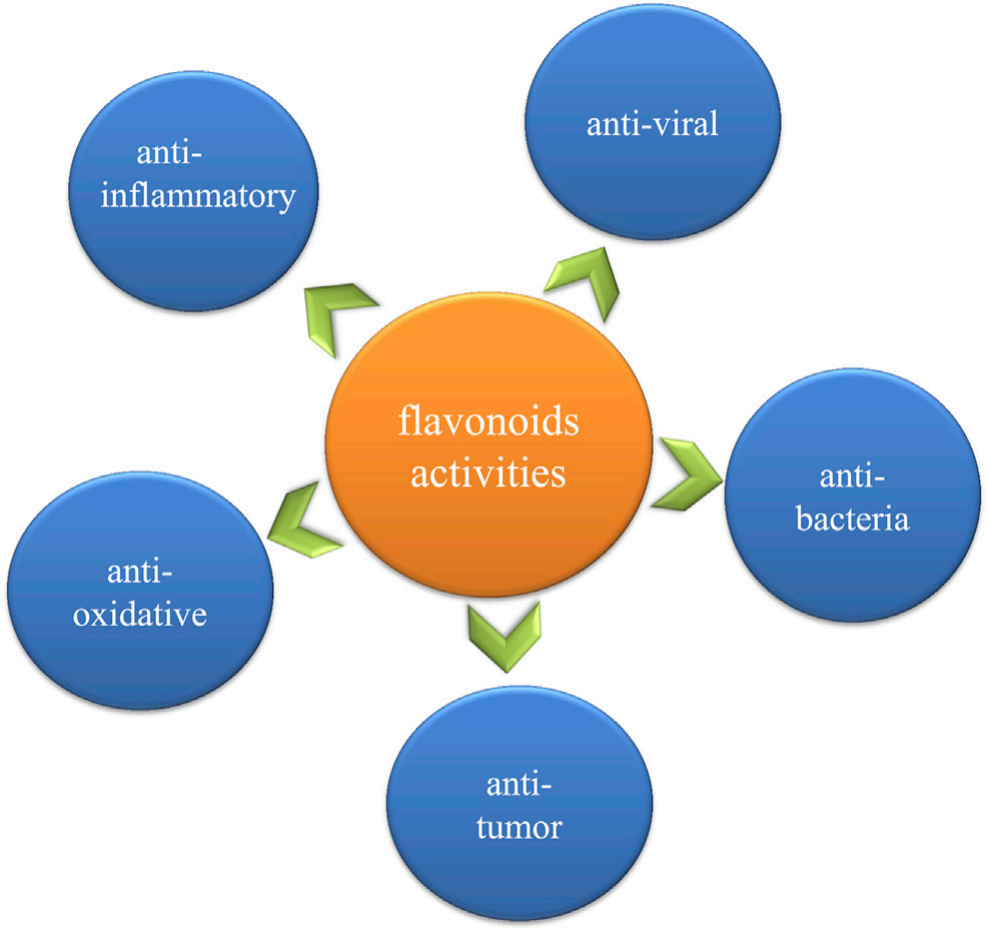
Schematic presentation of the biological activity of flavonoids.

Flavonoids have a wide range of pharmacological activities including anti-inflammatory, anti-oxidative, anti-microbial, and anti-tumor properties (Kumar and Pandey, 2013; Wen et al., 2017) (Figure 1). These properties are beneficial to bone regeneration. Firstly, many flavonoids, including baicalin (Guo et al., 2019; Huang et al., 2019), Kaempferol (Hwang et al., 2019), exert their anti-inflammatory effects by inhibiting the activation of the nuclear factor NF-κB pathway which is closely associated with inflammation. Kaempferol, a bioflavonoid extracted from *Persicaria tinctoria* (*Aiton*) *Spach* (Polygonaceae), prevented bone resorption through its anti-inflammatory property on osteoclast precursor cells (Hwang et al., 2019). Flavonoids have free radical scavenging activities through inhibiting the formation of free radicals, reducing lipid peroxidation, and stimulating antioxidant enzymes (Pietta, 2000). Secondly, given their anti-oxidative roles of flavonoids, some of them have been applied in clinical treatments. For example, troxerutin, a semi-synthetic flavonoid compound prepared by hydroxymethylation of rutin, is commonly used to treat ischemic cerebrovascular diseases, thrombophlebitis, central retinitis, and so on (Ahmadi and Mohammadinejad, 2021). Finally, flavonoids have been demonstrated anti-tumor effects through inhibiting tumor cell proliferation and metastasis, inducing tumor cell autophagy or apoptosis, and preventing tumor invasion. High intake of dietary flavonols, flavones and anthocyanidins may decrease the risk of cancer (Zamora-Ros et al., 2016; Chang et al., 2018).

Botanicals containing flavonoids compounds have been extensively used in traditional medicines for centuries, and nowadays many flavonoids have been extracted or synthesized and have been applied to treat various diseases in clinics. For example, diosmin, a semi-synthetic flavonoid drug with, is used to treat chronic venous insufficiency and varicose veins (Zheng et al., 2020). However, even if there are a number of well-tried treatment experiences of botanicals that are safe for clinical use, there are still many disagreements due to their ambiguous mechanisms. Investigating the underlying mechanisms of these herbal extracts will help gain deeper understanding of their beneficial pharmacological activities and facilitate medicinal applications.

## Effects of Flavonoids on Osteogenic Differentiation in of Mesenchymal Stem Cells

**FIGURE 2:**
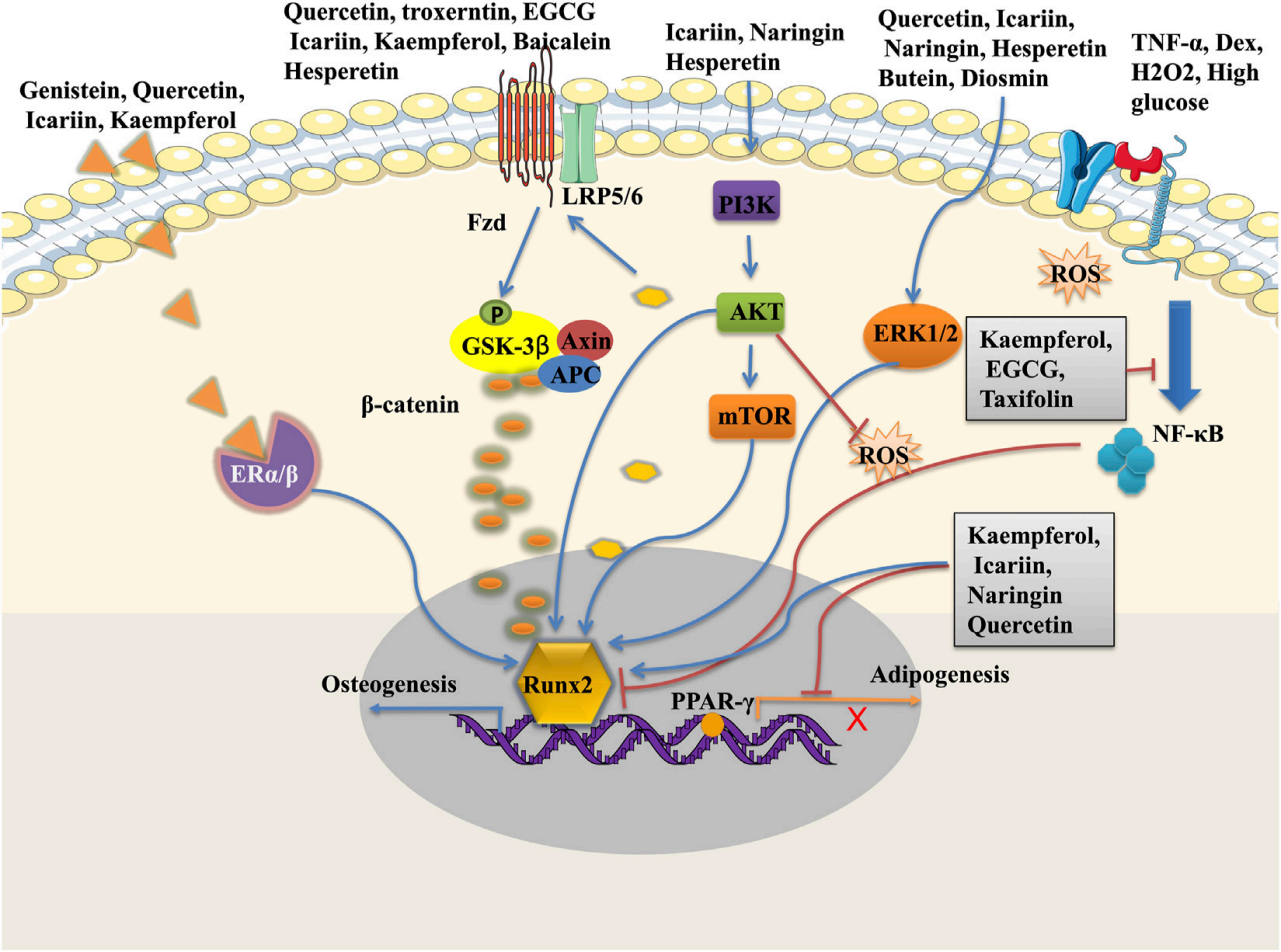
Signalling pathways of flavonoids in MSCs osteogenic differentiation.

Many herbal medicines for the treatment of fractures and other degenerative bone diseases have been used for several centuries in some countries, and modern pharmacology confirmed their major biologically active ingredients are flavonoids, such as soybean isoflavones, and flavonoids from *Drynaria roosii Nakaike (*Polypodiaceae*)*, *Epimedium brevicornu Maxim* (Berberidaceae), *etc.* (An et al., 2016). Among them, the corresponding active monomers, including naringin, icariin, genistein, and daidzein, have been proved to be able to regulate bone tissues metabolism by enhancing osteogenic differentiation and inhibiting osteoclast-mediated bone resorption. Especially their osteogenic induction potentials make flavonoids potential candidates to interfere with the osteogenic differentiation of MSCs (Table 2). Studies revealed flavonoids modulated the self-renewal and osteogenic differentiation potential of MSCs by targeting multiple signal pathways such as Wnt/β-catenin pathway, ERK pathway, PI3K/Akt pathway, and regulating the bone-specific markers and transcription factors including ALP, Runx2, BMP-2, Cbfa1, Osx (An et al., 2016; Zhang et al., 2016a) (Figure 2). In addition to directly stimulating the osteogenesis of MSCs, flavonoids could also indirectly affect the osteogenic differentiation of MSCs by their well-known antioxidant and anti-inflammatory properties (Schilling et al., 2014; Zhang ND. et al., 2016; Zhang L. et al., 2021). Besides, flavonoids were also loaded on bioscaffolds for the promotion of MSCs self-renewal and differentiation in bone regeneration. The utilization of flavonoids in biomaterials showed to be a great prospect for bone tissue engineering.

**TABLE 2:** The list of some flavonoids compounds on MSCs osteogenic differentiation.

Compound name	Dosage	*In vitro*	*In vivo*	Results and mechanism of action
Icariin	0.1–10 μM	hBMSC, hADSC	**-**	Enhance hBMSC and hADSC osteogenesis (Wu et al., 2017a)
	0.1 μM	rADSCs/glass scaffold	rat calvarial bone	Up-regulation VEGF expression, enhance angiogenesis, promote bone formation (Jing et al., 2018)
	5–40 μM	hBMSC	defect models	osteogenesis ↑, adipogenesis ↓; miR-23a ↓, active Wnt/β-catenin (Xu et al., 2021)
	0.01–1 μM	hBMSCs, rBMSCs	-	Osteogenesis ↑; sclerostin ↓, Wnt/β-catenin/ERα activation (Gao et al., 2021) (Wei et al., 2020)
	10–20 μM	rat mandibular MSCs	-	osteogenesis ↑, bone osteoporosis ↓; osteocalcin ↑, STAT 3 pathway activation (Xu et al., 2020)
	1 μM	rBMSCs	OVX rats	proliferation ↑, osteogenesis ↑, adipogenesis ↓; ERα pathway activation (Li et al., 2018)
	0.1–10 μM	rBMSCs	-	protect against iron overload induced dysfunction of BMSCs; active PI3K/AKT/mTOR pathway, inhibit ERK1/2 and JNK pathways (Yao et al., 2019)
	0.1 μM	rBMSCs	**-**	osteogenesis ↑, TAZ ↑; active ERα and Wnt/β-catenin pathway (Wei et al., 2017)
	50 mg/kg	mBMSCs	-	osteogenesis ↑, bone loss ↓; autophagy activation (Liang et al., 2019)
Quercetin	2–10 μM	hADSCs	**-**	proliferation ↓, osteogenesis ↑, ERK activity ↑, ER independent (Kim et al., 2006)
	1 μM	rBMSCs/nHA microspheres	OVX fracture rats	proliferation ↑, osteogenesis ↑, angiogenesis ↑; ERK, p38 and AKT activity ↑, RANKL ↓ (Zhou et al., 2017)
	10 μM	hBMSCs/scaffold	-	proliferation ↑, osteogenesis ↑, quercetin-crosslinked nHAp-modified decellularized goat-lung scaffold (Gupta et al., 2017)
	0.03 (wt%)	rabbit BMSC/SF/HAp scaffold	calvarial defect rats	osteogenesis ↑, proliferation ↑, bone regeneration ↑(Song and Tripathy, 2018)
	1–2 (wt%)	hUCMSCs/3D printing scaffold	-	cells growth and mitosis ↑, osteogenesis ↑, calcium deposit ↑(Huang et al., 2021)
	10 μM	rBMSC	-	osteogenesis ↑, adipogenesis ↓, active ERα-mediated circRNA-miR-326-5p-axis (Li et al., 2021)
Quercetin 3-O-β-D-galactopyranoside	1–25 μM	hBMSCs	-	proliferation ↑, osteogenesis ↑, adipogenesis ↓, active Wnt/BMP pathway, inhibit PPARγ pathways (Oh et al., 2020)
Isoquercitrin	0.1–1 μM	rBMSCs	maxillary expansion rats (10 mg/kg)	proliferation ↑, osteogenesis ↑, BMP2 ↑, bone formation ↑(Li et al., 2019a; Li et al., 2019b)
Hesperetin	1–10 μM	BMSCs	-	DEX-induced osteogenic inhibition ↓, active ERK signal pathway (Liu et al., 2021b)
	1 μM	hBMSCs/gelatin scaffold	rat osteotomy model	osteogenesis ↑, active ERK and Smad pathways, accelerate fracture healing (Xue et al., 2017)
	10–100 μM	PDLSCs	-	osteogenesis ↑, ROS ↓, active PI3K/Akt and β-catenin signal pathways (Kim et al., 2013)
Naringin	1–100 μg/ml	hAFSCs	-	proliferation ↑, osteogenesis ↑, BMP4 ↑, active Wnt/BMP pathway (Liu et al., 2017)
	0.03–0.1 (wt%)	hUCMSCs/SF-nHAp scaffolds	rabbit bone defect	proliferation ↑, osteogenesis ↑, angiogenesis ↑, bone regeneration ↑, active PI3K/Akt pathways (Zhao et al., 2021)
	20–100 μM	NPMSC	-	H2O2-induced cell apoptosis ↓; mitochondrial function ↑ (Nan et al., 2020)
	70 μg/ml	rabbit MSC/scaffolds	rabbit bone defect	bone formation ↑, inhibit BMPR-1A signaling (Dong and Ma, 2020)
	0.1 μM	rBMSC	-	restore TNF-α-induced osteogenesis and proliferation inhibition, p-IкBα and nuclear p65 ↓, inhibit NF-кB pathway (Cao et al., 2015)
	1–100 μg/ml	rBMSC	OVX rats	proliferation ↑, osteogenesis ↑, bone loss ↓, inhibit JAK2/STAT3 pathway (Wang et al., 2022)
Kaempferol	1 μM, 10 mg/kg	rBMSCs	OVX rats	bone density ↑, osteogenesis ↑, CXCL12 ↑, miR-10a-3p ↓(Liu et al., 2021a)
	0.1–100 μM or 25–100 mg/kg	rBMSCs	OVX rats bone defect	osteogenesis ↑, prevent OVX-induced osteoporosis, p-4E/BP1 ↓, p-S6K ↑, active mTOR pathway (Zhao et al., 2019)
	20–100 μM	rabbit BMSC	-	cells viability ↑, osteogenesis ↑, adipogenesis ↓, IL-10 ↑, IL6 ↓, inhibit NF-κB pathway (Zhu et al., 2017)
	50 μg/ml	rBMSC/TiO2 implants	rats femur bone defect	cell proliferation ↑, osteogenesis ↑, bone formation ↑, kaempferol-loaded TiO2 implants (Tsuchiya et al., 2018)
	2–10 μM	hADSCs	skull defect mice	cell proliferation ↓, osteogenesis ↑, ERK activity ↑, bone regenerating ability ↑(Kim et al., 2006)
EGCG	1–10 μM	hBMSCs	rats femoral bone defect	Osteogenesis ↑, Runx2 ↑, BMP2 ↑, bone defect healing ↑ (Lin et al., 2018b; Lin et al., 2019)
	5–40 μM	hBMSCs	-	hypoxia-induced apoptosis ↓, ameliorate hypoxia-induced osteogenesis reduction, miR-210 ↑, EFNA3 ↓(Qiu et al., 2016)
	1–10 μM	mBMSCs	-	cell proliferation ↓, osteogenesis ↑, Cbfa1 ↑, Runx2 ↑(Chen et al., 2005)
	1–10 μM	SCAPs	-	Proliferation ↑, osteogenesis ↑, Dspp ↑, Dmp-1 ↑, active BMP-Smad signaling pathway (Liu et al., 2021c)
	1–10 μM	rabbit BMSCs	nude mouse	EGCG/DC/HAp sponges increased cell internalization, attachment proliferation, ALP ↑ (Kook et al., 2018)
Genistein	0.01–1 μM	hBMSCs	-	Proliferation ↑, osteogenesis ↑, BMP2 ↑, SMAD5 ↑, RUNX2, ER dependent (Dai et al., 2013)
	5–20 μM	rBMSCs	-	Proliferation ↑, osteogenesis ↓, PPARγ ↑(Zhang et al., 2016a)
	1 μM	hBMSCs	-	Osteogenesis ↑, adipogenesis ↓, PPARγ ↓, ER-dependent, TGF-β ↑ (Heim et al., 2004)
Ipriflavone	0.4–0.8 μM	rBMSCs	OVX rats	osteogenesis ↑, osteoporosis ↓, BMD ↑ (Gao et al., 2018)
Malvidin	25 μM	hADSC	-	calcium deposits ↑, BMP-2 and Runx-2 ↑(Saulite et al., 2019)
Taxifolin	15 μM	hBMSC	-	Osteogenesis ↑, inhibit NF-κB pathway (Wang et al., 2017b)
Diosmin	10–100 μM	C3H10T1/2	-	Osteogenesis ↑, runx2 ↑, active FAK/ERK signaling pathway (Chandran et al., 2019)
Tricin	50–100 μM	hMSC(ATCC)	-	Proliferation ↑, osteogenesis ↑, Wnt3α- mediated (Zhang and Li, 2018)
Glabridin	5 μM	hBMSC	-	osteogenesis ↑, OCT4 gene↑(Heo and Lee, 2017)
HYSA	0.05–0.2 mg/ml	rabbit MSCs	-	prevent glucocorticoid-induced osteoporosis (Wan et al., 2014)
	0.1–0.5 mg/ml	rBMSCs/scaffold	rats bone defect	Osteogenesis ↑, HIF-1α ↑, BMP-2 ↑, new bone formation ↑(Deng et al., 2020)
Butein	1–30 μM	mBMSCs, hBMSCs	-	Osteogenesis ↑, adipogenesis ↓.activate ERK1/2 signaling pathway (Abdallah and Ali, 2020)
Baicalein	0.1–10 μM	TDSCs	tendon-bone healing rat model	Osteogenesis ↑, active Wnt/β-catenin signaling pathway (Tian et al., 2018)
Amentoflavone	0.1–5 μM	hBMSCs	-	Osteogenesis ↑, p-p38 ↑, active JNK and p38 MAPK pathway (Zha et al., 2016)
Troxerutin	25–200 μM	hBMSC	fracture rats model	Osteogenesis ↑, fracture healing ↑, active Wnt/β-catenin signaling (Yang et al., 2021)
Fisetin	200–800 μg/ml	rBMSCs/BC scaffold	-	BC scaffold loaded with fisetin promote osteogenesis (Vadaye Kheiry et al., 2018) proliferation ↓, migration ↓, YAP ↓, osteogenic differentiation ↓(Lorthongpanich and Charoenwongpaiboon, 2021)
	1–30 μM	chorion tissue hMSC	-	BC scaffold loaded with fisetin promote osteogenesis (Vadaye Kheiry et al., 2018) proliferation ↓, migration ↓, YAP ↓, osteogenic differentiation ↓(Lorthongpanich and Charoenwongpaiboon, 2021)

hUCMSCs, human umbilical cord-derived mesenchymal stem cells; hAFSCs, human amniotic fluid-derived stem cells; NPMSC, nucleus pulposus-derived mesenchymal stem cells; NG/SF/HAp, naringin-inlaid composite silk fibroin/hydroxyapatite; SCAPs, Stem cells from apical papilla; TDSCs, tendon-derived stem cells; PDLSCs, periodontal ligament stem cells; HYSA, Hydroxy Safflower Yellow A.

### Icariin

5Icariin (ICA) is a kind of flavonol glycoside and generally extracted from *Epimedium brevicornu Maxim* (Berberidaceae), a traditional Chinese herbal medicine for bone repair. A large number of studies have revealed that ICA had protective roles on bone loss and bone regeneration (Fu et al., 2016; Wang J. et al., 2016; Wei et al., 2017; Ye et al., 2017; Wang et al., 2018; Liang et al., 2019; Gao et al., 2021). ICA not only increased the ALP activity and mineralization of BMSCs but also reduced bone resorption mediated by overactivated osteoclasts in OVX-induced osteoporosis mice (Liang et al., 2019). In addition, ICA has been shown to promote proliferation by activating the Wnt/β-catenin signaling pathway (Wang J. et al., 2016; Gao et al., 2021), which is the most important pathway in osteogenesis. In Sprague-Dawley (SD) rats, ICA stimulated BMSCs proliferation by increasing the phosphorylation level of GSK-3β and cyclin D1 protein (Fu et al., 2016). ICA has been reported to promote proliferation and osteogenic differentiation through increasing the expression of transcriptional coactivator with PDZ-binding motif (TAZ) both in rat BMSCs and ADSCs (Wei et al., 2017; Ye et al., 2017). Furthermore, the up-regulation of TAZ induced by ICA could be blocked by ICI 182780 or DKK1 (the Wnt/β-catenin pathway inhibitor), which indicated Wnt/β-catenin signaling pathway and ER signaling pathway were involved in the osteogenic differentiation of rBMSCs induced by ICA. The proliferation of rat BMSCs enhanced by ICA is also achieved through activating ERK and p38 MAPK signaling (Fu et al., 2016).

Similarly, Icariside II, a kind of metabolite of ICA, enhanced osteogenic differentiation of BMSCs by increasing ALP activity and calcium deposition at 10 µM (Luo et al., 2015). Icaritin, another metabolic product of ICA, significantly increased ALP activity and calcium deposition at concentrations 0.1–10 µM in human BMSCs and ADSCs through increasing the expression of BMPs, and showed better osteogenesis induction ability than rhBMP-2 (Wu T. et al., 2017).

### Quercetin and Kempferol

Quercetin and kaempferol are the main representatives of flavonols, which are the 3-hydroxy derivatives of flavanones, probably the most ubiquitous subclass of flavonoids in nature. The anti-inflammatory and antioxidant effects of quercetin and kaempferol have been repeatedly reported (Babaei et al., 2018; Dabeek and Marra, 2019; Kashyap et al., 2019).

With the development of tissue engineering, the roles of quercetin on the osteogenic differentiation of MSC gradually attracted more attentions. It was reported quercetin could increase bone mineral density (BMD) and improve bone biomechanical properties in postmenopausal osteoporosis rat models (Yuan et al., 2018). The increase of BMP-2 and TGF-β1, two main osteogenic factors, was observed in rat and mouse BMSCs treated with quercetin (Li et al., 2015). Furthermore, the ability of quercetin to stimulate proliferation and osteogenic differentiation of mouse BMSCs could be blocked by estrogen receptor inhibitor ICI182780 (Pang et al., 2018). This indicates that quercetin enhances osteogenic differentiation of MSCs by an estrogen receptor-dependent mechanism. However, in another study similar effects of quercetin on human adipose tissue-derived stem cells (hADSCs) could not reversed by ICI182780, despite the fact that it up-regulated the expression of BMP2, Runx2, as well as activated ERK phosphorylation (Kim et al., 2006). Quercetin also promoted the differentiation and proliferation of BMSCs through inhibiting NF-κB activation and β-catenin degradation stimulated by TNF-α (Yuan et al., 2018). Bian et al. also observed Wnt/β-catenin pathway activation played an important role in the osteogenic differentiation of quercetin treated-BMSCs (Bian et al., 2021). Quercetin stimulated osteogenic differentiation of BMSCs by increasing connexin 43 expressions (Zhang et al., 2020) which could enhance osteogenic differentiation of BMSCs by promoting GSK-3β/β-catenin signaling pathways (Lin FX. et al., 2018).

Kaempferol, another representative flavonol, had a similar osteogenic induction potential with quercetin in periodontal ligament stem cells (PDLSCs). The treatment with 10^–6^ M kaempferol increased cell viability, ALP activity, and enhanced calcium mineralization of PDLSCs. Furthermore, these effects of kaempferol could be reversed by XAV939, a tankyrase inhibitor, indicating Wnt/β-catenin signaling pathway was involved (Nie et al., 2020). The activated Wnt/β-catenin signaling by kaempferol, to some extent, depended on estrogen receptors, as the activation of Wnt/β-catenin could be markedly blocked by the ICI 182780, the inhibitor of estrogen receptors (Sharma and Nam, 2019).

### Naringin and Hesperetin

Naringin and hesperetin are two of the best-studied compounds in flavanones (Den Hartogh and Tsiani, 2019; Tutunchi et al., 2020). Naringin is rich in grapefruit and also the main active component of *Drynaria roosii Nakaike* (Polypodiaceae), a Chinese herbal medicine commonly used to treat orthopedic disorders and bone injury (Lavrador et al., 2018). Naringin dose-dependently increased ALP activity and Alizarin red S staining, and decreased PPARγ2 mRNA expression that is the marker of adipogenesis in rat BMSCs under osteogenic induction. Furthermore, this osteogenic effect of naringin could be reversed by the inhibitor of Notch signaling, indicating naringin exerted its role through activating the Notch signaling pathway (Yu G. Y. et al., 2016). In human BMSCs, wang et al. demonstrated naringin promoted proliferation and osteogenesis by activating the ERK signaling pathway (Wang H. et al., 2017). The gene expressions of bone morphogenetic protein 4 (BMP4), runt-related transcription factor 2 (Runx2), β-catenin, and Cyclin D1 were significantly up-regulated by naringin in human amniotic fluid-derived stem cells (Liu et al., 2017). In addition, Naringin alleviates the inhibitory effect of various stimulating factors on osteogenic differentiation of MSC. In a glucocorticoid-induced osteoporosis rat model, naringin not only improved bone mineral density and bone morphology parameters, but also stimulated the expression of autophagy-related factors including Beclin-1 and p62, which indicated autophagy was also involved in the bone protective effect of naringin (Ge and Zhou, 2021). Similarly, Hesperidin and its aglycone, hesperetin, two flavonoids from citrus species, also exerted protective roles in the osteogenesis of MSCs (Parhiz et al., 2015). In dexamethasone-treated BMSCs, the inhibition of MSC osteogenesis was reversed by the intervention of hesperetin through activating the ERK signal pathway (Liu L. et al., 2021). retreatment with 1–100 μM concentration hesperetin significantly increased the osteogenic activity of periodontal ligament stem cells under high glucose conditions. It was considered scavenged intracellular ROS produced and activated PI3K/Akt and β-catenin signaling pathway by hesperetin were responsible for this protective effect (Kim et al., 2013).

### Epigallocatechin-3-Gallate

Tea is abundant in flavonoids, mainly including catechins, theaflavins, alkaloids, etc., (-)-epigallocatechin-3-gallate (EGCG) is the major catechin isolated from *Green Tea* (Friedman, 2007). As an antioxidant and anti-inflammatory agent, EGCG plays an important role in maintaining the balance of bone metabolism through the inhibition of bone resorption as well as the enhancement of bone formation (Nishioku et al., 2020). Although EGCG alone could not induce osteogenic differentiation of MSC, EGCG was able to enhance osteogenesis under osteogenic induction environment through upregulating BMP2 expression (Jin et al., 2021). Lin et al. showed EGCG enhanced osteogenic differentiation at the concentrations range from 1 to 10 µM both in murine and human BMSCs by increasing the expression of osteoblastic genes including BMP2, Runx2, ALP, osteonectin, and osteocalcin, as well as promoting ALP activity and mineral deposits (Lin S. Y. et al., 2018). Furthermore, the effect of EGCG on promoting the mineralization of human MSCs is independent of its antioxidant activity (Lin S. Y. et al., 2018). In human ADSCs, 5 μM EGCG significantly enhanced cell proliferation and ALP activity, experimental data revealed that osteo-inductive effects of EGCG on human ADSCs were associated with the inhibition of adipogenesis-related gene expression (Zhang et al., 2019). For the stem cells from apical papilla (SCAPs), at low concentrations, EGCG promoted the cell proliferation and increased ALP activity as well as mineral deposition through activating the BMP-Smad Signaling Pathway (Liu Z. et al., 2021). In addition to promoting osteogenic differentiation directly, EGCG treatment significantly reversed the inhibition of MSC osteogenesis induced by hydrogen peroxide and inflammatory cytokines. EGCG could enhance osteogenic differentiation by increasing the expression of β-catenin and cyclin D1 in H2O2-induced human BMSCs (Wang D. et al., 2016). A similar protective effect of EGCG was also observed on TNF-α-induced osteogenesis inhibition of MSC, in which EGCG reversed the TNF-α-induced destructive through inhibition of NF-κB signaling (Liu et al., 2016).

### Genistein

Genistein is one of the most abundant isoflavones in *Glycine max* (*L.*) *Merr* (*Fabaceae*), and it is also called a phytoestrogen owing to its similar structure to that of human estrogen. It could bind to ERα and ERβ and exert ER-mediated estrogenic effects including increasing bone formation and repressing adipose tissue (Jaiswal et al., 2019) At the same time, it possessed antiestrogenic effects as well as non-ER-mediated effects like inhibiting tyrosine kinase (Dang et al., 2003). Genistein exerted estrogenic effects mainly by binding to ER α, even with a stronger affinity to ERβ than to ERα, genistein, and 8-prenylgenistein (a prenylated derivative), all of them could inhibit GSK-3β enzymatic activities though inducing GSK-3β phosphorylation at Serine-9 in human BMSCs and murine pre-osteoblast MC3T3-E1 cells. In addition, 8-prenylgenistein showed stronger osteogenic ability than genistein in MC3T3-E1 cells by increasing ERα-dependent β-catenin protein expression (Qiu et al., 2020). It seemed that both Wnt/β-catenin and ERα-associated signaling were involved in the osteogenic activities of genistein. Owing to its well-known estrogenic ability, genistein directly or indirectly affected the osteogenic and adipogenic differentiation of MSCs. In the early stages of differentiation of human primary BMSCs osteogenic markers were strongly up-regulated by genistein, while during adipogenic differentiation, adipogenic regulators, including PPARγ and CCAAT/enhancer-binding protein-α, were down-regulated after genistein treatment (Heim et al., 2004). A lineage shift from adipogenesis to osteogenesis induced by genistein was observed in murine MSCs and pre-osteoblasts isolated from newborn mice (Li et al., 2005). However, in another study, genistein was reported to enhance adipogenesis of human MSCs and suppressed their osteogenesis through regulating the expression of PPARγ (Zhang LY. et al., 2016). These contradictory results may be caused by the dose of genistein, at low concentrations (≦1 μM), genistein acted like estrogen, stimulating osteogenesis and inhibiting adipogenesis, whereas at high concentrations (>1 μM), genistein acted as a ligand of PPARγ, leading to up-regulation of adipogenesis and down-regulation of osteogenesis (Dang et al., 2003).

### Other Flavonoids

In addition to the flavonoids mentioned above, other flavonoid extracts like baicalein (Ren et al., 2021), apigenin (Pan et al., 2021), amentoflavone (Zha et al., 2016), and anthocyanins (Saulite et al., 2019) have also been found to enhance osteogenic differentiation of MSCs. In human periodontal ligament cells (hPDLCs), baicalein induced osteogenic differentiation dose-dependently (1.25–10 μM) by activating the Wnt/β-catenin signaling pathway (Chen et al., 2017). Cyanidin-3-O-glucoside (C3G), the most common type of anthocyanin in nature, was shown to increase the expression of osteoblastic markers and osteoblast proliferation rate both in mouse MC3T3-E1 cells and human osteoblasts (extracted from the hip joint of patients with osteoporosis) by regulating ERK1/2 signaling pathway (Hu et al., 2021).

### Inhibitory Effects of Flavonoids on Mesenchymal Stem Cells Osteogenic Differentiation

Although most studies showed that flavonoids promoted the osteogenic differentiation of MSC, some reports showed that flavonoids sometimes had an inhibitory effect on the osteogenic differentiation of MSC, and promoted adipogenesis (Hu et al., 2011; Zhang LY. et al., 2016; Casado-Díaz et al., 2016). Some flavonoids showed that they promoted the adipogenesis and inhibited osteogenesis of MSCs (Hu et al., 2011; Casado-Díaz et al., 2016; Lorthongpanich et al., 2021). Two isoprenylated flavonoids isolated from the twigs of *Morus alba L* (*Moraceae*; *Morus alba L*) significantly promoted adipogenesis and induced up-regulation of the expression of adipocyte-specific genes, aP2 and GLUT4 in 3T3L1 cells (Hu et al., 2011). In another study showed that high concentration of quercetin inhibited osteoblastic differentiation and promoted adipogenesis through Wnt/β-catenin inhibition. Which indicate such possible adverse effects of high use concentrations should be taken into account in nutraceutical or pharmaceutical strategies using flavonoids (Casado-Díaz et al., 2016).

## The Applications of Flavonoids in Bone Disorders by Promoting Osteogenic Differentiation of Mesenchymal Stem Cells

The effects of flavonoids on bone defects had been extensively established using animal models (Yu et al., 2021; Zhao et al., 2021; Zhou and Xie, 2021). Flavonoids stimulated bone formation by increasing cell viability, matrix mineralization, calcium deposition, and up-regulation of osteogenic genes (Wu Y. et al., 2017; Preethi Soundarya et al., 2018). Meanwhile, flavonoids have great importance in treating bone disorders owing to their anti-inflammatory and anti-oxidative activities as we described above. Many flavonoids have been widely used in ovariectomized (OVX) osteoporotic, age-related osteoporotic models as well as glucocorticoid-induced osteoporosis, by regulating osteoblast-regulated bone formation and/or osteoclast-mediated bone resorption.

The impaired capability of osteogenic differentiation and senescence of MSCs are important pathogeneses of osteoporosis caused by various reasons (Jiang et al., 2021). In the aging process, as well as in glucocorticoid-induced osteoporosis, the ability of MSCs’ commitment towards the osteogenic lineage is impaired, while the adipogenesis is increased. Reactivating the osteogenic differentiation ability of MSCs is considered an important way to osteoporosis treatment. Icariin was effective in preventing postmenopausal osteoporosis through stimulating osteogenic differentiation of BMSCs (Wang et al., 2018), and it also protected against glucocorticoid-induced osteonecrosis of the femoral head in rats (Huang et al., 2018). Hesperetin alleviated glucocorticoid-induced inhibition of osteogenic differentiation through ERK signal pathway in BMSCs (Liu L. et al., 2021). Flavonoids also have great potential for the treatment of diabetes-induced osteoporosis owing to their anti-oxidative and adipogenesis inhibition activities (Nelson-Dooley et al., 2005; Kawser Hossain et al., 2016). Diabetes-induced osteoporosis is caused by chronic hyperglycemia, advanced glycated end products, and oxidative stress (Mohsin et al., 2019). In a rat model of diabetic osteoporosis, icariin could prevent diabetic osteoporosis by reducing blood glucose, inhibiting bone marrow adipogenesis, as well as up-regulation the expression of Runx2 and OPG (Qi et al., 2019).

MSC-based cellular therapy is a promising novel therapeutic strategy for osteonecrosis of the femoral head (ONFH). Flavonoids can increase bone formation in femoral heads by promoting MSCs proliferation and osteogenic differentiation. In methylprednisolone-induced rat ONFH models, the lithium chloride treatment group displayed a higher vessel volume and better trabecular structures as well as more OCN expression compared with methylprednisolone group, MSCs extracted from rats treated with lithium chloride had higher proliferative and osteogenic ability (Zhang Y. L. et al., 2021). Zefeng Yu et al (2016) also demonstrated lithium could enhance angiogenesis and stabilize osteogenic/adipogenic balance in glucocorticoid-induced ONFH rat models by activating the β-catenin pathway.

## Combination Use of Flavonoids and Biomaterials in Bone Tissue Engineering

Mesenchymal stem cells combined with biological scaffold materials loaded with flavonoids are an excellent option for the application of flavonoids in the field of bone tissue engineering repair, and incorporation of flavonoids into biomaterials or scaffolds has been proved as a reliable technology for bone tissue regeneration, For example, the quercetin/silk fibroin/hydroxyapatite scaffolds with BMSCs increased the formation of new collagenous tissue and tissue ingrowth in a rat calvarial defect model, quercetin was found to promote cell proliferation and osteogenic differentiation of BMSC cultured in scaffolds *in vitro* (Song and Tripathy, 2018). Flavonoids also can stabilize collagen and inhibit its degradation in biological systems (Shavandi et al., 2018). The BMSCs-laden quercetin/collagen/hydroxyapatite sponge was proved as an alternative biomaterial for bone regeneration (Song et al., 2020). Kaempferol-immobilized titanium dioxide promotes the formation of new bone and is considered an effective tool for bone regeneration around dental implants (Tsuchiya et al., 2018). Promoting the proliferation and osteogenic differentiation of MSCs on scaffolds is the main role of flavonoids in the construction of bone tissue engineering. Besides, flavonoids enhance bone regeneration by counteracting the negative effect of oxidative stress on MSCs viability and differentiation (Forte et al., 2016; Chu et al., 2018). However, recent developments in bone tissue engineering focusing on flavonoids and their potent biological properties that enhance bone health has been well-reviewed (Preethi Soundarya et al., 2018). The potential of a combination of biomaterials loaded with flavonoids and MSCs might be enormous in bone tissue engineering.

## Future Prospective

Extensive evidence showed the roles of flavonoids in regenerative and therapeutic medicine. Flavonoids as stimulants significantly affect the proliferation and osteogenic differentiation of MSCs. To further effectively screen and evaluate the application potential of flavonoids in bone tissue engineering and repair, it is very necessary to establish a standard and effective osteogenic differentiation protocol of MSC induced by flavonoids. Furthermore, the dose-effect relationship between MSCs and flavonoids should also be well established to achieve desired effects and reduce side effects. Given most flavonoids compounds are not having good solubility and low hydrophilicity, delivery systems, such as nanocarriers, with flavonoids are promising strategies for the improvement of cell uptake efficiency. In addition, MSCs combined with biological scaffold materials loaded with flavonoids are an excellent option for the application of flavonoids in the field of bone tissue engineering.

## Conclusion

Flavonoids have a wide range of pharmacological activities and widely exist in nature. Flavonoids play a crucial role in the bone repair process not only through direct induction of osteoblastic differentiation, but also through their anti-inflammatory and anti-oxidant effects. MSCs combined with flavonoids are a promising alternative in stem cell therapy and bone tissue engineering construction. Flavonoids can help to increase proliferation and osteogenic differentiation of MSCs as well as modulate the microenvironment in the injured bone. To promote their clinical use, more works need to be done to improve their safety, efficacy, and quality, and to explore the mechanisms underlying their roles.
